# Postoperative short-term use of the nonsteroidal anti-inflammatory drug flurbiprofen did not increase the anastomotic leakage rate in patients undergoing elective gastrointestinal surgery—a retrospective cohort study

**DOI:** 10.1186/s13741-022-00275-y

**Published:** 2022-08-19

**Authors:** Huang Nie, Yiming Hao, Xiangying Feng, Lina Ma, Yumei Ma, Zhen Zhang, Xi’an Han, Jian zhong Zhang, Ping Zhang, Qingchuan Zhao, Hailong Dong

**Affiliations:** 1 Department of Anesthesiology, Xijing Hospital, Air Force Medical University, Xi’an, China; 2 Department of Gastrointestinal Surgery, Xijing Hospital, Air Force Medical University, Xi’an, China; 3 The Unimed Scientific, Inc., Wu Xi, China

**Keywords:** Nonsteroidal anti-inflammatory drugs, Flurbiprofen, Gastrointestinal surgery, Anastomotic leakage

## Abstract

**Background:**

Flurbiprofen has been one of the most commonly used nonsteroidal anti-inflammatory drugs (NSAIDs) in China and other Asian countries for perioperative multimodal analgesia in recent years, yet its association with anastomotic leakage in gastrointestinal anastomoses is unknown. The current study was designed to investigate whether short-term administration of flurbiprofen would increase the risk of anastomotic leakage in patients undergoing gastrointestinal surgery for cancer resection.

**Methods:**

A total of 3653 patients (2487 (66.1%) men) undergoing elective operation for gastrointestinal cancer between 18 July 2017 and 30 Oct 2020 were included. The median age was 61 years (interquartile range 53–67 years). The exposure was the short-term postoperative use of flurbiprofen (defined as flurbiprofen treatment within the first week after surgery). The primary outcome was the frequency of clinical anastomotic leakage.

**Results:**

Of 3653 patients with available data who were included in the final analysis, 2282 received flurbiprofen administration, and 1371 did not. Anastomotic leakage was not significantly increased among the patients receiving flurbiprofen compared with those who did not (1.62% v 1.46%; *P*=0.70). In-hospital mortality was comparable between the two groups (0.04% v 0.07%; *P*=0.72). After adjusted analysis, male sex (OR 3.51, 95% CI 1.80–6.85), ASA score of 3–4 (OR 2.69, 95% CI 1.62–4.48), and intraoperative infusion (OR 2.24, 95% CI 1.19–4.21) were identified as risk factors for anastomotic leakage.

**Conclusions:**

Postoperative short-term use of flurbiprofen did not increase the risk of anastomotic leakage in gastrointestinal anastomoses.

## Introduction

Anastomotic leakage following gastrointestinal surgery is a potentially serious complication resulting in increased postoperative morbidity and mortality. The reported leakage rates are approximately 3% after colonic resections and 10% after rectal resections, with mortality rates of up to 32% (Guenaga et al. 2009; Choi et al. 2006). For gastric cancer patients undergoing gastrectomy, the leakage rate was reported to be 2.7–15% (Haga et al. 2011; Inokuchi et al. 2015).

In the past two decades, NSAIDs have been strongly recommended by the Enhanced Recovery After Surgery Society (Nygren et al. 2012; Mortensen et al. 2014) as important multimodal analgesic components for various surgeries; thus, the possible effect of nonsteroidal anti-inflammatory drugs (NSAIDs) on the risk of anastomotic leakage has been increasingly studied. Retrospective studies have shown an association between anastomotic leakage and postoperative treatment with NSAIDs (Klein et al. 2012; Klein et al. 2009; Gorissen et al. 2012). Although animal studies have suggested that reduced collagen production and microthromboses resulting from cyclooxygenase inhibition might explain the adverse effects of NSAIDs, their anti-inflammatory effects may be beneficial after surgery. Controversy remains about whether and which type (nonselective or cyclooxygenase-2 selective) of NSAIDs are associated with anastomotic leakage (Jamjittrong et al. 2020).

Currently, the nonselective NSAID flurbiprofen is widely used during the early postoperative period in most hospitals in China and other Asian countries. However, no investigation has studied whether short-term administration of flurbiprofen increases the risk of anastomotic leakage after gastrointestinal surgery. To investigate the possible effect of postoperative flurbiprofen treatment on patients undergoing gastrointestinal surgery, we performed a study based on data from the “Real World Study of Enhanced Recovery After Surgery Program” of a tertiary teaching hospital, which included detailed information on perioperative treatment.

## Materials and methods

### Patients and study design

This study was based on data from the “Real World Study of Enhanced Recovery After Surgery Program” of a tertiary teaching hospital. We aimed to compare the risk of anastomotic leakage among patients receiving flurbiprofen, which is the most commonly used NSAID in our hospital, with those not receiving regular NSAID treatment. With the electronic recording systems, all treatments administered at our hospital were documented.

This retrospective cohort study enrolled consecutive patients from a tertiary hospital between 18 July 2017 and 30 Oct 2020. Patients were deemed eligible for inclusion if they were scheduled for elective gastrointestinal surgery for gastrointestinal cancer and received a primary anastomosis. The exclusion criteria were (1) patients receiving more than one type of NSAID; (2) patients receiving NSAIDs other than flurbiprofen; or (3) patients receiving flurbiprofen for more than 7 days after surgery. This study was approved by KY20202116-C-1 from Approval Form I E C of our hospital. The report was written in accordance with the STROBE Statement (Vandenbroucke et al. 2014). The study was reported in line with the STROCSS criteria (Agha et al. 2019).

### Data collection and definition

Data pertaining to baseline demographics, medical interventions, and clinical outcomes were obtained prospectively and automatically saved in the database prior to analysis. The system we used for data collection and derivation was developed by Unimed Scientific, Inc. (Wu Xi, China). From the database, we retrieved information on demographic variables, alcohol and tobacco use, comorbidities (pre-existing diabetes mellitus, ischemic heart disease, respiratory system disease, or hypertension), procedure type, open or laparoscopic procedure, tumor T stage, intraoperative blood loss (mL) and transfusion (whether it occurred or not), and anastomotic leakage. We defined the relevant daily dose as at least 50 mg and maximal dose of 200 mg for flurbiprofen. Anastomotic leakage was defined as leakage detected symptomatically, radiologically, or during surgery (Matthiessen et al. 2007).

### Statistical analysis

Data are shown as medians and interquartile ranges for continuous variables and frequencies and proportions for categorical variables. Between-group differences were assessed via a two-tailed Student’s *t* test (for parametric variables) or the Mann-Whitney *U* test (for nonparametric variables). Categorical variables were analyzed via the chi-square test, CMH-chi-square test, or Fisher’s exact test as appropriate. To identify possible risk factors for anastomotic leakage, we planned to perform univariate logistic regression analyses on all variables with less than 10% missing data. These variables included NSAID use, intraoperative transfusion, colorectal section or gastrectomy, sex, age at time of operation, intraoperative blood loss, American Society of Anesthesiologists score, open or laparoscopic surgery, and tumor T stage. We included all variables with *P*<0.2 in a multivariate logistic regression analysis, and we also performed stepwise regression analysis (method: backward, likelihood ratio). We presented the results as odds ratios (ORs) and 95% confidence intervals (CIs) and *P* values. Differences between independent proportions were calculated as absolute risk increases with confidence intervals and calculated according to the method in reference (Newcombe 1998). Statistical tests were interpreted at a two-sided significance level of 5%. All statistical analyses were performed using SAS version 9.3 (SAS Institute, Inc., Cary, NC, USA).

## Results

### Study participants and data completeness

**Fig. 1:**
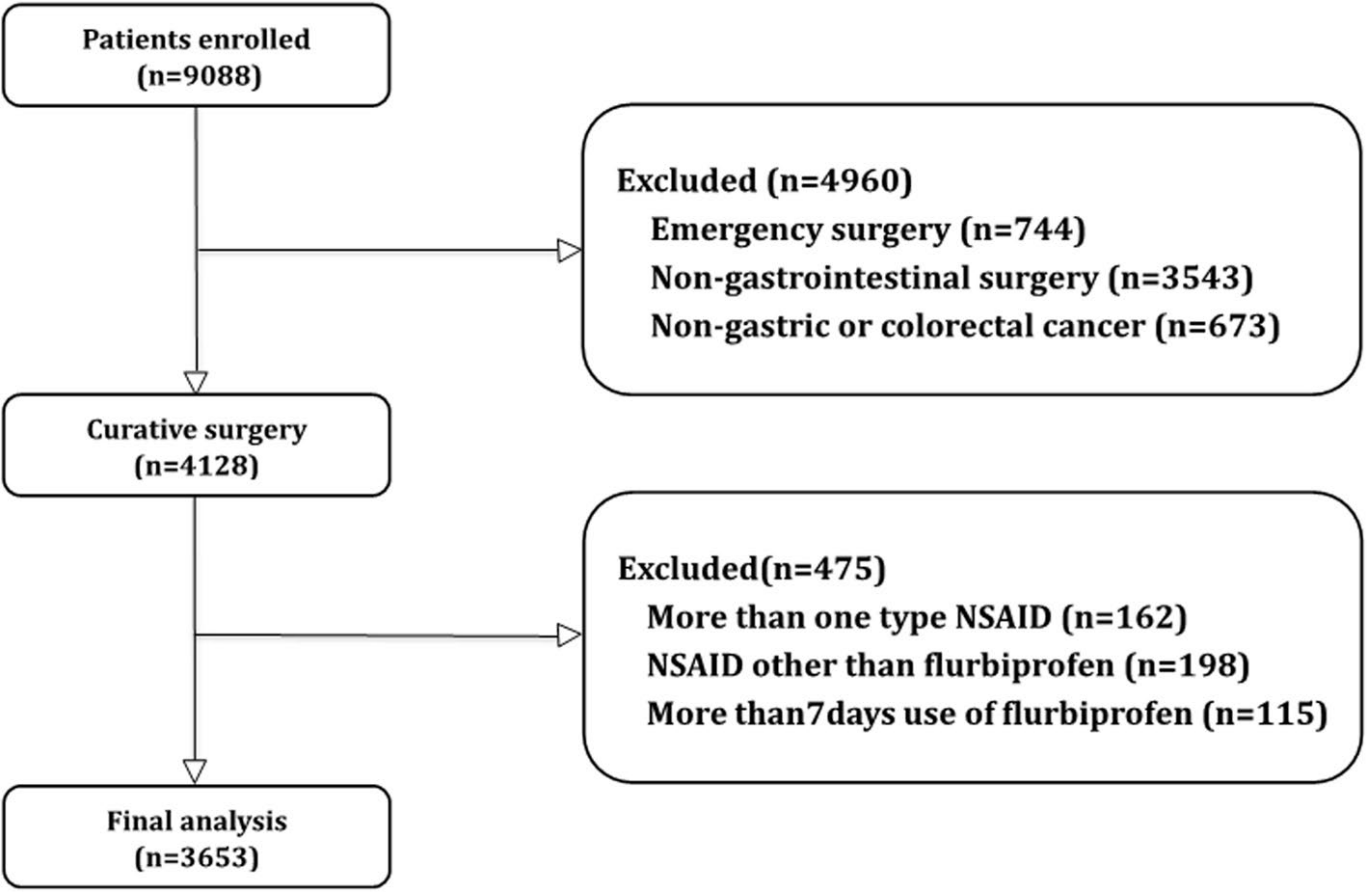
Flow chart of patients included in the study. NSAID, nonsteroidal anti-inflammatory drug

Based on the inclusion criteria, we retrieved data for 4128 patients with elective gastrointestinal cancer resection and primary anastomosis from our surgical database (Fig. 1). Of these, 198 patients were excluded for administration of NSAIDs other than flurbiprofen, 162 patients were excluded for two types of NSAID use, and 115 were excluded for more than 7 days of administration. After exclusion of these patients, 3653 remained for final analysis. In this group, 2282 (62%) patients received postoperative treatment with flurbiprofen, and 1371 (38%) did not receive NSAIDs. Of all the variables, tobacco use and alcohol had approximately 30% missing data, whereas others’ completeness was above 99%.

### Patient demographic and clinical details

Table 1 presents the demographic variables and data completeness. The median age of the entire study population was 61 years (interquartile range 53–67 years), and 2487 (68.1%) were men. A total of 2054 patients underwent curative gastrectomy for gastric cancer, and 1599 underwent colorectal surgery for colorectal carcinoma. We found 1708 (47%) laparoscopic procedures. Overall, 57 patients had anastomotic leakage, and 2 (0.05%) died in the hospital. There were more female patients in the flurbiprofen group than in the control group. We observed more patients with ischemic heart disease in the flurbiprofen group. Patients who received flurbiprofen had more laparoscopic procedures and more colorectal surgeries. Less intraoperative blood loss was found, and intraoperative transfusion was less frequent in patients receiving flurbiprofen (Table 2).

**Table 1:** Population characteristics and data completeness

	Total study populations (*n*=3653)	Missing data (%)
Age	61 (53–67)	0
Sex		
Male	2487 (68.08%)	0
Female	1166 (31.92%)	
BMI	23 (20.9–25.1)	0.27
Tobacco use		
Non-smoker	1532 (60.58%)	30.19
Previous smoker	842 (33.02%)	
Active smoker	176 (6.90%)	
Alcohol		
Non-drinker	1822 (71.65%)	30.38
Previous drinker	613 (24.10%)	
Active drinker	108 (4.25%)	
Ischemic heart disease	129 (3.53%)	0
Hypertension	757 (20.72%)	0
Diabetes	338 (9.25%)	0
Respiratory system disease	31 (0.85%)	0
ASA score		
1	66 (1.81%)	0.05
2	3160 (86.55%)	
3	411 (11.26%)	
4	14 (0.38%)	
Tumor stage (pTNM)		
**1**	947 (25.92%)	0
2	1138 (31.15%)	
3	1541 (42.18%)	
4	27 (0.75%)	
Procedure type		0
Gastrectomy	2054 (56.22%)	
Colorectomy	1599 (43.77%)	
Procedure approach		
Laparoscopic	1708 (46.76%)	0
Open	1945 (53.24%)	
Intraoperative blood loss (ml)	100 (50–150)	0.19
Intraoperative transfusion	249 (6.82%)	0
Operating duration	3 (2.42–3.83)	0
Anastomotic leakage	57 (1.56%)	0
In-hospital mortality	2 (0.05%)	0

**Table 2:** Population characteristics and surgical factors according to NSAID use

	Flurbiprofen (***n***=2282)	No NSAIDs (***n***=1371)	*P* value
Age	61 (53–67)	61 (54–68)	0.2759
Sex, *n* (%)			0.03
Male	1524 (66.78%)	963 (70.24%)	
Female	758 (33.22%)	408 (29.76%)	
BMI	23 (20.9–25)	23 (20.9–25.1)	0.7447
Tobacco use			0.3813
Non-smoker	952 (60.33%)	580 (59.67%)	
Previous smoker	514 (22.57%)	328 (33.74%)	
Active smoker	112 (7.10%)	64 (6.58%)	
Alcohol			0.7876
Non-drinker	1123 (71.44%)	699 (71.99%)	
Previous drinker	386 (24.55 %)	227 (23.38%)	
Active drinker	63 (4.01%)	45 (4.63%)	
Ischemic heart disease	93 (4.08%)	36 (2.63%)	0.0215
Hypertension	481 (21.08%)	276 (20.13%)	0.4943
Diabetes	213(9.93%)	125 (9.12%)	0.8269
Respiratory system disease	19 (0.83%)	12 (0.88%)	0.8917
ASA score			0.7537
1	44 (1.93%)	22 (1.61%)	
2	1964 (86.10%)	1196 (87.3%)	
3	264 (11.57%)	147 (10.73%)	
4	9 (0.39%)	5 (0.36%)	
Tumor stage			0.4766
1	574 (25.15%)	373 (27.21%)	
2	717 (31.42%)	421 (30.71%)	
3	972 (42.59%)	569 (41.50%)	
4	19 (0.83%)	8 (0.58%)	
Procedure type			<.0001
Gastrectomy	1222 (53.55%)	832 (60.69%)	
Colorectomy	1060 (46.45%)	539 (39.31%)	
Procedure approach			<.0001
Laparoscopic	1256 (55.04%)	452 (33.09%)	
Open	1026 (44.96%)	919 (66.91%)	
Intraoperative blood loss (ml)	100 (50–150)	100 (50–200)	<.0001
Intraoperative transfusion (yes)	140 (6.13%)	109 (7.95%)	0.035
Operating duration	3 (2.42–3.83)	3 (2.42–3.75)	0.809
Anastomotic leakage	37 (1.62%)	20 (1.46%)	0.701
In-hospital mortality	1 (0.04%)	1 (0.07%)	0.7156

*BMI* body mass index, *ASA* American Society of Anesthesiologists

### Anastomotic leakage in relation to flurbiprofen use

In total, 57 (1.56%) patients had a symptomatic anastomotic leak. According to ISREC’s grading system (Kulu et al. 2013), 1 patient was graded A, 27 (47.4%) patients were graded B, and 29 (50.9%) patients were graded C. The leaks were diagnosed on postoperative day 4 at a median (interquartile range 3–6 days). Anastomotic leakage occurred in 37 (1.62%) patients treated with flurbiprofen and in 20 (1.36%) controls. With regard to postoperative flurbiprofen treatment, no increased risk was found (*P*=0.70). To identify individual risk factors for anastomotic leakage, based on less than 10% missing data, we performed univariate logistic regression analyses (Table 3). Based on the *P*<0.2 limit, we included sex, intraoperative transfusion, ASA score, and tumor stage in the multivariate analysis (Table 4). The analysis showed a significantly increased risk of anastomotic leakage in males (odds ratio 3.51, (95% confidence interval 1.58 to 7.78); *P*=0.002) and in those with an ASA score higher than 2 (odds ratio 2.67, (95% confidence interval 1.45 to 4.92); *P*=0.0016). Additionally, we performed stepwise regression analysis (Table 5). Male sex (odds ratio 3.51 (95% confidence interval 1.80 to 6.85); *P*=0.002), ASA score higher than 2 (odds ratio 2.69 (95% confidence interval 1.62 to 4.48); *P*=0.0014) and intraoperative transfusion (odds ratio 2.24 (95% confidence interval 1.19 to 4.21); *P*=0.0365) were associated with an increased risk of anastomotic leakage.

**Table 3:** Risk factors of anastomotic leakage based on univariate logistic regression analysis

	Odds ratio (95% CI)	*P* value
NSAIDs use (F vs No)	1.11 [0.64–1.93]	0.7012
Intraoperative Transfusion (yes vs no)	2.62 [1.27–5.41]	0.0091
Sex (male vs female)	3.40 [1.54–7.52]	0.0025
Age (≥65 vs <65)	0.98 [0.57–1.70]	0.9438
ASA score (≥3 vs <3)	3.04 [1.69–5.46]	0.0002
Procedure type (colorectal vs gastric)	0.87 [0.51–1.48]	0.6000
Procedure (open vs laparoscopic)	0.79 [0.47–1.33]	0.3713
Tumor stage (4 vs 3 vs 2 vs 1 )	1.39 [0.99–1.94]	0.0561

*NSAIDs* non-steroidal anti-inflammatory drugs, *ASA* American Society of Anesthesiologists

**Table 4:** Risk factors of anastomotic leakage based on multivariate regression analysis

	Odds ratio (95% CI)	*P* value
Intraoperative transfusion (yes vs no)	2.09 [0.98–4.46]	0.0576
Sex (male vs female)	3.51 [1.58–7.78]	0.0020
ASA score (≥3 vs <3)	2.67 [1.45–4.92]	0.0016
Tumor stage (4 vs 3 vs 2 vs 1 )	1.34 [0.95–1.88]	0.0911

*ASA* American Society of Anesthesiologists

**Table 5:** Risk factors of anastomotic leakage based on stepwise regression analysis

	Odds ratio (95% CI)	*P* value
Intraoperative transfusion (yes vs no)	2.24 [1.19–4.21]	0.0365
Sex (male vs female)	3.51 [1.80–6.85]	0.0020
ASA score (≥3 vs <3)	2.69 [1.62–4.48]	0.0014

*ASA* American Society of Anesthesiologists

## Discussion

To our knowledge, this was the first large retrospective study to investigate whether short-term administration of flurbiprofen increases the risk of anastomotic leakage after elective gastrointestinal surgery. Our results showed that postoperative administration of flurbiprofen within 1 week did not increase the anastomotic leakage rate in patients undergoing elective gastrointestinal surgery for carcinoma resection.

First, the strength of this study is the large number of patients included, ensuring high statistical power in the analyses. Second, unlike most retrospective studies that only covered colorectal surgery, our study focused on both upper and lower gastrointestinal tract surgery. Since NSAIDs are often used in analgesic regimens to spare opioids in such circumstances (McDaid et al. 2010; Zhang et al. 2011), our results are relevant to daily clinical practice. Moreover, patients’ NSAID exposure was completely and reliably recorded, thus minimizing the risk of misclassification. Finally, our study investigated the association of regularly used flurbiprofen (widely used in China and other countries) and anastomotic leakage (Zhang et al. 2011; Nishina et al. 2000; Sultan et al. 2009). To some extent, the result could alleviate the worries about using flurbiprofen for multimodal analgesia in this group of patients.

A limitation of this study is that it is not population-based, thereby allowing the possibility of selection bias. In addition, as a retrospective observational study, this report is vulnerable to bias and confounding. However, we adjusted the results by multivariate logistic regression, as is recommended to reduce confounding.

The association between NSAIDs and anastomotic leakage has been evaluated in many studies; however, the different types of NSAIDs and leak definitions make comparisons difficult. Although several retrospective studies have indicated an increased risk of leakage after NSAID treatment, a number of more recent studies presented similar results as ours (Klein et al. 2009; Gorissen et al. 2012). In Washington State’s Surgical Care and Outcomes Assessment Program (SCOAP), data from colorectal and bariatric surgery indicated that any NSAID use within 24 h following surgery did not increase the risk of leakage. Saleb et al. investigated the effect of the nonselective agent ketorolac on anastomotic leakage and found no increase in risk after treatment (Saleh et al. 2014). In a Swedish retrospective multicenter cohort study, NSAID use after anterior resection for rectal cancer did not increase the risk of anastomotic leakage (Kverneng Hultberg et al. 2017). In the stepwise regression analysis, we identified risk factors for anastomotic leakage. Male sex and blood transfusion have been suggested to increase the risk of anastomotic leakage both in colorectal and gastric anastomosis (Iversen et al. 2008; Lipska et al. 2006; Alves et al. 2002; Golub et al. 1997; Mäkelä et al. 2003). These findings correspond well with the literature and thus confirm the validity of our data. We also found that an ASA score higher than 2 was a risk factor that has never been reported by other studies.

In a rat colonic anastomosis model, flurbiprofen-treated rats had higher collagen levels than those of the control or prostaglandin E2 group, suggesting that inhibition of prostaglandin synthesis by administration of flurbiprofen may improve healing in the colon (Brennan et al. 1984). Similarly, in a newly published animal study, the author did not find evidence that diclofenac or ketorolac increases the leakage risk of colocolic anastomoses (Ghiselli et al. 2020). These results may explain the present study finding that short-term use of flurbiprofen does not seem to harm the healing of anastomoses. In our study, intraoperative transfusion was associated with an odds ratio of 2.24 for anastomotic leakage (Table 5). This result is expected, since intraoperative transfusion is a surrogate marker for a difficult procedure, a suboptimal surgical technique, or perhaps an insufficient anastomotic perfusion caused by anemia or hypotension. An ASA score higher than 2 was also identified as a risk factor, and we could not rule out other indirect factors that might be affected.

## Conclusion

Postoperative short-term use of flurbiprofen did not increase the risk of anastomotic leakage in gastrointestinal anastomoses. However, a well-designed randomized clinical trial is warranted to verify the results of this observational study.

## Data Availability

The datasets generated during and/or analyzed during the current study are not publicly available due to statutory provisions regarding data and privacy protection.
